# Supporting the early use of echocardiography in blunt chest trauma

**DOI:** 10.1186/2036-7902-4-7

**Published:** 2012-05-03

**Authors:** Scott B Jennings, Jonathan Rice

**Affiliations:** 1 The Canberra Hospital, Canberra, Australian Capital Territory, 2606, Australia; 2 Moruya District Hospital, Moruya, New South Wales, 2537, Australia

**Keywords:** Blunt chest trauma, Echocardiography, Ultrasound, Cardiac tamponade, Cardiogenic shock.

## Abstract

This case reports a very unusual mechanism of cardiac rupture following an episode of multiple blunt chest trauma. The patient, a professional jockey, was trampled by horses, and although shocked on hospital admission, he did not present with signs and symptoms that were consistent with cardiogenic shock. This case highlights the difficult and subjective nature of clinical examination in emergency situations when dealing with cases of acute cardiac tamponade. It further emphasises the lack of sensitivity of traditional trauma imaging and investigative approaches such as the standard anteroposterior chest X-ray and electrocardiogram. The diagnosis of acute cardiac tamponade was not made until tertiary-care-centre arrival, when ultrasound technology in the form of bedside echocardiography was used, facilitating emergency surgery to repair a ruptured left ventricle. It is hoped that the sharing of this case will alert fellow clinicians to this uncommon but possible mechanism of cardiac rupture and subsequent tamponade, encourage the early use of echocardiography at the bedside in hypotensive blunt chest trauma cases and reinforce the principles of the Advanced Trauma Life Support course in treating trauma victims.

## Background

Trauma is the third leading cause of death in the under-40 age group in the United States, with blunt force trauma being a very common mechanism. For example, in the United States, 25% of all fatalities have arisen from a form of chest trauma in general [1]. More specifically, chest injury has been cited as the most important injury of polytraumas with an incidence of 45% to 65% and an associated mortality of up to 60% [2]. Blunt chest trauma is a common result of impact and deceleration injuries such as motor vehicle accidents, falls, crush injuries, occupational exposures and sporting hazards [1-5]. Incidentally, blunt chest trauma is more common than penetrating chest trauma in the United States [1]. Cardiac rupture following blunt chest trauma is an exceedingly uncommon consequence but is associated with an extremely high mortality [5].

The first repair of a cardiac rupture following blunt trauma occurred in 1955; however, only a paucity of series reviewing the outcomes post-rupture have been undertaken since this initial case [1]. Mortality from cardiac rupture following blunt chest trauma has been reported to be as high as 81.3% to 85% [6,7]. A 5-year study of blunt cardiac rupture cases revealed that of the 32 cases reviewed, 20 presented in cardiac arrest (non-survivors), and of those who presented with vital signs, only 6 survived [5]. Another retrospective study of 160 autopsy cases showed that of the 40 fatalities from blunt chest trauma, approximately 5 % made it to the hospital alive, with the majority dying at the scene (86%) or during retrieval (approximately 8%) [4]. Similar figures of 80% early death have been reported in similar studies [1]. Shorr et al also reported a 100% (39/39) unsuccessful resuscitative thoracotomy rate in presentations without vital signs [1]. Cardiac injury distribution following blunt chest trauma was widely varied in two large retrospective studies reviewed. Right atrial rupture accounted for 5% and 40.6%, left atrial rupture 10% and 25%, right ventricular rupture 15% and 31.3% and left ventricular rupture 10% and 12.5%, respectively [4,5]. However, the literature reviewed described only three cases of cardiac rupture involving horses, with one case being mentioned by two separate reviews [1,5]. No outcome regarding the twice-mentioned case was described. The second report, emphasising the use of post-mortem computed tomography (CT) imaging, described the fatal case of a 13-year-old female who collapsed after being kicked by a pony. Resuscitative attempts were unsuccessful and the diagnosis was confirmed at post-mortem CT imaging, revealing a 3-cm laceration of the left ventricle causing cardiac tamponade [1,5,6]. The third case, a 23-year-old female, survived after suffering a 3-cm laceration to the right atrium. This patient exhibited early classic signs of cardiac tamponade and was initially treated with pericardiocentesis and pericardial catheter insertion until definitive repair with oversew via sternotomy could be performed [8].

Hence, the case described here is significant for a number of reasons. Firstly, there are only three cases of individuals suffering a cardiac rupture following horse kicks in the literature, but none from trampling. Horse riding is undoubtedly a popular recreational and occupational activity worldwide; thus, emergency medical personnel should be extremely mindful when dealing with chest injuries in these individuals. Secondly, a common misconception about the postulated mechanism for rupture is not the kinetic energy applied to the thorax per se, but the direct compression of the heart between the sternum and dorsal spine [9], hence the mechanism that occurred in this case: trampling effect of the horses [9,10]. Thirdly, the use of chest X-rays in the diagnosis of cardiac tamponade can be extremely unreliable, especially in the acute setting. For example, a study of the diagnostic value comparing chest X-ray and echocardiography for pericardial effusions in post-operative cardiac patients showed a positive radiological diagnosis in 20 % compared to the 86 % using ultrasonography. This diagnosis rate of 20 % was also with the added benefit of previous films for comparison [11]. Fourthly, the role of electrocardiography (ECG) in blunt cardiac trauma has been reported as both non-specific and as evidence for evolving cardiac damage. ECG, therefore, is not diagnostic of acute cardiac tamponade but may be of assistance in making the diagnosis [12,13].

## Case presentation

**Figure 1:**
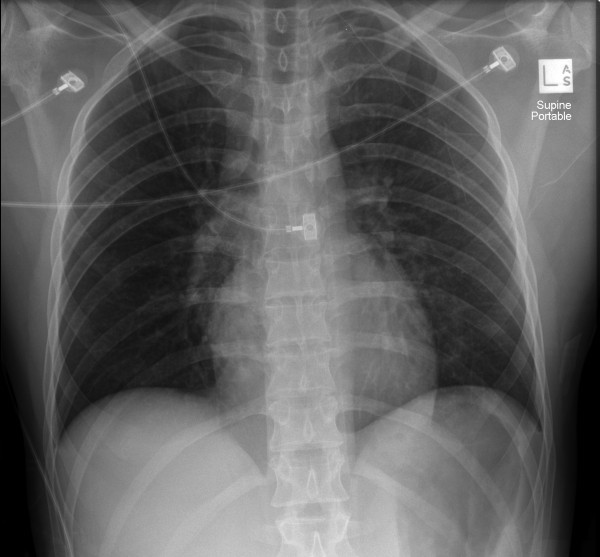
Initial anteroposterior chest X-ray.

These images assist in reporting an unusual case of ventricular rupture following an episode of blunt chest trauma. This case is of a 38-year-old male professional jockey who was undertaking track work when he fell, with following horses trampling him. Ambulance response was <10 min, and the patient had an initial Glasgow coma scale (GCS) of 3 and unrecordable blood pressure. After receiving basic trauma care onsite, he was brought to the district hospital Emergency Department. GCS had now improved to 14 and a blood pressure of 60/40 mmHg was recorded. Physical examination at this stage revealed no abnormalities except for a ‘hoof’ mark on the left lateral lower chest wall and cool peripheries. The initial anteroposterior chest X-ray appeared and was reported as normal, although ‘globular’ was used to describe the cardiac shadow (Figure 1). An ECG performed at this stage showed a sinus rhythm of 93/min with ventricular premature complexes, aberrant SV complexes and ST elevation in leads V2-V4.

**Figure 2:**
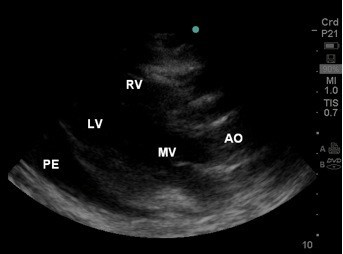
**Emergency Department B-mode echocardiogram showing a large pericardial effusion (PE).** LV, left ventricle; RV, right ventricle; MV, mitral valve; AO, aorta.

Resuscitation included volume (crystalloid) and pharmacological (metaraminol) interventions; however, blood pressure continued to fall (47/25 mmHg, invasive monitoring) with the pulse paradoxically slowing (60/min). The patient was then retrieved to a tertiary centre, having muffled heart sounds and a tender, distended abdomen on arrival. An urgent bedside echocardiogram revealed a large pericardial effusion, acute cardiac tamponade (Figure 2).

The echocardiographic features of pericardial effusion in this case was a large volume of pericardial fluid, right atrial and ventricular diastolic collapse and a distended inferior vena cava (IVC) secondary to right-sided heart failure (IVC image not shown). Doppler features can also demonstrate changes in trans-mitral and tricuspid flows seen with inspiration and expiration and flow pattern changes in the superior and inferior vena cava [14]. Subxiphoid/subcostal and transthoracic windows are usually first obtained to demonstrate pericardial effusion size and to determine the best pericardiocentesis needle trajectory [15]. After echocardiographic diagnosis, the patient was immediately taken for bilateral salvage, anterolateral thoracotomies and exploratory laparotomy. A large haemopericardium was evacuated secondary to a ruptured left ventricle. Laparotomy (with re-look) was not significant, and the patient remained in the Intensive Care Unit for 6 days. The patient continued to improve and was discharged home soon after.

The noteworthy aspects of this case are as follows. Firstly, this case highlights the difficultly in diagnosing acute cardiac tamponade post-blunt trauma at the bedside by using conventional films. Secondly, multi-traumas may have ongoing haemorrhage from another identified or unidentified source, masking a cardiac tamponade as an injury or complimentary mechanism of shock [8]. For example, 30% of individuals who have suffered a ventricular rupture may also have a concurrent pericardial rupture causing exsanguination into the hemithorax [8]. Thirdly, this case is consistent with other blunt chest trauma reports with two-thirds of cardiac rupture cases showing no other signs of major injury [8,9].

The initial chest X-ray (Figure 1) is also of particular interest as it shows an essentially normal anteroposterior film with perhaps a globular or enlarged cardiac silhouette, a feature noted more clearly in other reports [16]. However, the difficultly in determining acute cardiac sizing when only a single anteroposterior film is used and how the increased sensitivity of echocardiography immediately clinched the diagnosis must again be acknowledged.

Fourthly, the patient became paradoxically bradycardic with an acute cardiac tamponade. Tachycardia is the normal response to a decrease in stroke volume secondary to acute cardiac tamponade. Two postulated mechanisms may explain the findings seen in this case. It has been postulated that bradycardia can arise from direct compression of the vagus nerve or recurrent laryngeal nerves or as a direct consequence of extensive myocardial contusions [13,17,18]. Bradycardia in this case could have included a significant element of both postulated mechanisms. It would be reasonable to deduce that as the pericardial effusion increased in size, it caused compression of either nerve system resulting in bradycardia [18]. Improved neurological status in this patient would also have removed brainstem injury as a potential source of bradycardia [18]. Echocardiography also has the ability to identify regional cardiac wall abnormalities, further aiding the clinician in determining overall cardiac function, especially when extensive myocardial contusions are suspected [19]. However, cardiac lacerations/perforations with an intact pericardium in a patient that survives to reach the hospital are not routinely seen on echocardiography. Once again, the presence of a pericardial effusion, mechanism of injury and clinical status should suffice in aiding the clinician in making the diagnosis [20].

## Conclusions

This case strongly supports the early use of echocardiography in blunt chest trauma with echocardiography being especially useful in the prompt and accurate diagnosis of cardiac tamponade [12,16]. Rozycki et al. reported in a series of 1,540 patients with truncal injuries that echocardiography is most accurate when used for the evaluation of patients with precordial or transthoracic wounds with possible haemopericardium and for the immediate evaluation of hypotensive patients with blunt chest trauma [21]. In this series, echocardiography yielded a sensitivity of 100% and a specificity of 99.3% for patients with precordial or transthoracic wounds [21]. The use of ultrasound in the acute trauma setting may also be useful to triage patients by idenitfying intra-thoracic injuries earlier (M Kot, unpublished). Also, early combination of normal echocardiogram and ECG has been shown to reduce the number of intensive care admissions for cardiac monitoring post-blunt chest trauma. It is hoped that this case will alert fellow clinicians to maintain a high index of suspicion for cardiac tamponade in hypotensive blunt chest traumas, encourage the early use of echocardiography with these injuries and reinforce the principles of systematic evaluation and treatment as taught in the Advanced Trauma Life Support course.

## Consent

Written informed consent was obtained from the patient for publication of this report and any accompanying images. A copy of the written consent is available for review by the Editor-in-Chief of this journal.

## Competing interests

The authors declare that they have no competing interests.

## Authors' contributions

Both author's contributed equally to the preparation of this manuscript. All authors read and approved the final manuscript.

## Authors' information

Dr. Scott B Jennings, BSc MBBS (Hons), is a surgical registrar, at The Canberra Hospital, Canberra, Australian Capital Territory, Australia. Dr. Jonathan Rice, MBBS (Adel) FRACS FRCS (Eng), is a consultant general surgeon at Moruya District Hospital, Moruya, New South Wales, Australia.
